# Sensory-Modality Preferences Among Undergraduate Medical Students at a North Indian Medical College: Implications for Online and Blended Teaching

**DOI:** 10.7759/cureus.117233

**Published:** 2026-09-30

**Authors:** Angel, Aastha, Kailash Chander, Deepshika Kamra, Girish Joseph, Neena Bhatti

**Affiliations:** 1 Anatomy, Jaipur National University, Jaipur, IND; 2 Anatomy, Christian Medical College and Hospital, Ludhiana, IND; 3 Community Medicine, Christian Medical College and Hospital, Ludhiana, IND; 4 Pharmacology and Therapeutics, Christian Medical College and Hospital, Ludhiana, IND

**Keywords:** kinesthetic learning, medical education, online learning, sensory-modality preferences, vark

## Abstract

Background

The abrupt shift to online instruction during the COVID-19 pandemic renewed interest in how individual learners prefer to absorb and process information. The visual, aural, read/write, and kinesthetic (VARK) model offers a simple, self-report description of a learner's sensory-modality preferences and can inform how teaching is delivered.

Objective

The objective of this study is to describe the VARK sensory-modality preferences of undergraduate bachelor of medicine, bachelor of surgery (MBBS) students at a North Indian medical college and explore whether these preferences differed by sex and MBBS batch.

Materials and methods

In this cross-sectional study, 298 undergraduate medical students completed version 8.01 of the 16-item VARK questionnaire. Responses were scored with the standard VARK algorithm, and students were classified as unimodal (single preference) or multimodal (bimodal, trimodal, or quadrimodal). Category proportions were compared between the sexes using the chi-square test or Fisher's exact test, with significance set at p<0.05.

Results

Overall, 154 students (51.7%) were multimodal and 144 (48.3%) were unimodal. Kinesthetic learning dominated the unimodal group, accounting for 112 students (77.8% of unimodal learners; 37.6% of the whole sample), whereas read/write was the least common single preference (five students; 1.7%). The most frequent bimodal, trimodal, and quadrimodal patterns were AK (42; 14.1%), VAK (23; 7.7%), and all-four VARK (60; 20.1%), respectively. Mean modality scores were highest for kinesthetic (9.03) and lowest for read/write (3.82). Female students showed a higher proportion of multimodal learners than male students (57.2% vs. 45.3%; Fisher's exact p=0.048), although the four-category distribution and mean modality scores did not differ significantly by sex.

Conclusions

Students at this college showed a near-even distribution between unimodal and multimodal sensory-modality preferences, with kinesthetic preference being the most common single-modality preference. These findings describe the sensory-modality preferences reported by the cohort but do not demonstrate that any particular teaching modality is superior or more effective. Further studies linking sensory-modality preferences with educational outcomes are needed.

## Introduction

The COVID-19 pandemic accelerated a rapid, worldwide move to online instruction. Students who would ordinarily have attended lectures in person found themselves learning through a screen, and teachers who had taught face-to-face had to reshape their delivery for a digital setting. This transition raised a persistent concern among educators and learners alike: whether the same quality of education could be maintained once the classroom was replaced by an online environment [1].

Online, or electronic, learning uses network and internet technologies to exchange information between teacher and learner and has become a mainstay of higher education, including medical education. Engagement in online settings tends to differ from that in the classroom; learners often feel more detached from peers and instructors and more dependent on their own initiative, and those with limited technical support can feel overwhelmed. Understanding how to help students learn effectively in this environment is therefore increasingly important [2]. Recent evidence from medical and dental students has similarly highlighted the widespread use of electronic resources and a preference for combining them with conventional teaching rather than using them as a replacement, supporting the rationale for balanced online and blended educational approaches [3].

One way to approach this question is to characterize how learners prefer to receive information. A learning preference may be understood as the manner in which a learner prefers to take in, process, and recall new information. The VARK model, introduced by Fleming and colleagues, focuses specifically on the sensory channel or channels a learner favors: visual (V; diagrams, charts, and symbolic representations), aural (A; listening and discussion), read/write (R; text-based input and note-taking), and kinesthetic (K; hands-on, experiential learning) [4,5]. Other frameworks describe related preferences, including Kolb's experiential learning model [6], Honey and Mumford's learning-styles model [7], and Felder and Silverman's model [8]. Because most everyday learning draws on more than one channel, learners with a single dominant preference are described as unimodal and those with more than one as multimodal - bimodal, trimodal, or quadrimodal [4].

VARK has been applied widely in medical education. Studies of medical students commonly report a preponderance of multimodal learners and, frequently, a strong kinesthetic component [9-11]. Knowledge of a cohort's modality profile can help teachers diversify how they present material and can prompt students to broaden their own study strategies. The interpretation of learning styles is, however, contested. Systematic reviews have found little or no evidence that matching instruction to a learner's preferred style improves outcomes - the so-called meshing hypothesis and several authors caution against organizing teaching around style labels [12-15]. In this study, we therefore treat VARK as a descriptive tool for understanding a cohort and for encouraging varied, active teaching, not as a prescription for individually style-matched instruction.

Data on the modality preferences of Indian medical undergraduates remain limited, and local profiles may differ from those reported elsewhere because of differences in prior schooling and assessment culture. Understanding these preferences in a local medical student population may provide a descriptive basis for future educational research, particularly in the context of increasing use of online and blended learning. Previous studies have also reported inconsistent findings regarding sex-related differences in learning preferences [10,16-18]. In addition, students at different stages of undergraduate medical education may have different learning experiences and educational exposure, potentially influencing their reported preferences. We therefore examined sensory-modality preferences by sex and explored differences across the bachelor of medicine and bachelor of surgery (MBBS) cohorts. Sex was examined as an exploratory demographic variable because previous studies have reported inconsistent findings regarding sex-related differences in learning preferences [10,16-18]. Male and female students at our institution participate in the same academic and clinical learning environment; therefore, sex was not considered a proxy for differences in curriculum or teaching exposure. Against the backdrop of expanded online teaching, we assessed the VARK preferences of undergraduate medical students at our institution using the standard VARK questionnaire [4]. The objective was to identify the preferred sensory modality or modalities of these students and to explore whether preferences differed by sex or MBBS batch, thereby providing a descriptive profile that may inform future educational research on online and blended teaching.

## Materials and methods

Study design and setting

This was a cross-sectional, questionnaire-based study conducted among undergraduate MBBS students at Christian Medical College and Hospital, Ludhiana, a teaching hospital in North India. Data were collected over a six-month period from October 2020 to March 2021. The study is reported in accordance with the STROBE (Strengthening the Reporting of Observational Studies in Epidemiology) guidance for cross-sectional studies [19].

Participants

Undergraduate MBBS students enrolled at the college during the study period were eligible to participate. Students who gave informed consent were included; questionnaires that were incompletely filled were excluded. All eligible students who were available during the study period were invited, so sampling was by complete enumeration (census) of the accessible cohort rather than by random selection, and no formal sample-size calculation was performed. A total of 299 students were approached, all of whom completed and returned the questionnaire (response rate 100%). One questionnaire was incomplete and was excluded, leaving 298 questionnaires for analysis. Students were drawn from the MBBS batches of 2017, 2018, 2019, and 2020. MBBS batch was recorded to permit exploratory comparison of sensory-modality preferences across stages of undergraduate medical education.

Instrument

Version 8.01 of the 16-item VARK questionnaire was used (copyright held by Neil D. Fleming, Christchurch, New Zealand, and Charles C. Bonwell, Green Mountain Falls, Colorado, United States), with permission obtained from the developer [4]. The VARK questionnaire (Version 8.01) is a 16-item self-report instrument designed to identify learners’ preferences across four sensory modalities: visual (V), aural (A), read/write (R), and kinesthetic (K). Each item presents a learning-related situation and offers four response options corresponding to these modalities. Participants may select more than one option when applicable, allowing identification of multimodal preferences. For scoring, each selected response was mapped to its corresponding VARK category using the standard scoring chart provided with the questionnaire. The number of responses assigned to each category was summed to obtain separate V, A, R, and K scores for each participant. The resulting scores were interpreted to describe the participant’s predominant or multimodal sensory-modality preference.

Each item presents everyday scenarios with response options mapped to the visual, aural, read/write, or kinesthetic modalities; respondents may select more than one option per item, reflecting the multimodal nature of real-world learning.

Procedure

Students were briefed about the study and completed the questionnaire during regular working hours. For each respondent, the V, A, R, and K totals were entered into a spreadsheet (Microsoft Excel; Microsoft Corp., Redmond, WA). Each response set was scored using the standard VARK scoring algorithm. Students were classified as unimodal when the standard VARK algorithm identified a single dominant sensory-modality preference and as multimodal when two or more modalities met the algorithm’s criteria for a preference. No investigator-defined cut-off was applied. Higher-order categorization (bimodal, trimodal, and quadrimodal) was carried out using the VARK result-analysis service for researchers [4]. Students with two, three, or four identified preferences were classified as bimodal, trimodal, or quadrimodal, respectively.

Statistical Analysis

Sensory-modality categories and preferences were summarized as counts and percentages, and modality scores as means with standard deviations. Proportions were compared between sexes using the chi-square test with Yates’ continuity correction when expected cell frequencies were adequate; Fisher's exact test was used for the 2×2 unimodal versus multimodal comparison as an exact alternative. Differences in categorical distributions across MBBS batches were assessed using the chi-square test. Modality scores across the four MBBS batches were compared using the Kruskal-Wallis test, with Holm-adjusted pairwise Mann-Whitney U tests performed when the overall comparison was significant. No additional adjustment for multiple comparisons was applied to the exploratory sex-wise analyses; these analyses were interpreted cautiously and considered hypothesis-generating. A two-sided p-value <0.05 was considered statistically significant.

Ethical Considerations

The study was conducted in accordance with the principles of the Declaration of Helsinki. It was reviewed and approved by the Institutional Research Committee of Christian Medical College, Ludhiana (reference number CMC/3140; approved September 16, 2020). Written informed consent was obtained from all participants, and confidentiality was maintained throughout; only the participant's sex and MBBS batch were recorded alongside the questionnaire responses, and confidentiality was maintained throughout.

## Results

Complete data were available for 298 undergraduate medical students, of whom 159 (53.4%) were women and 139 (46.6%) were men. Overall, 154 students (51.7%) were multimodal and 144 (48.3%) were unimodal, a near-even split. Within the multimodal group, 59 students (19.8%) were bimodal, 35 (11.7%) were trimodal, and 60 (20.1%) were quadrimodal (Figure 1).

**Figure 1:**
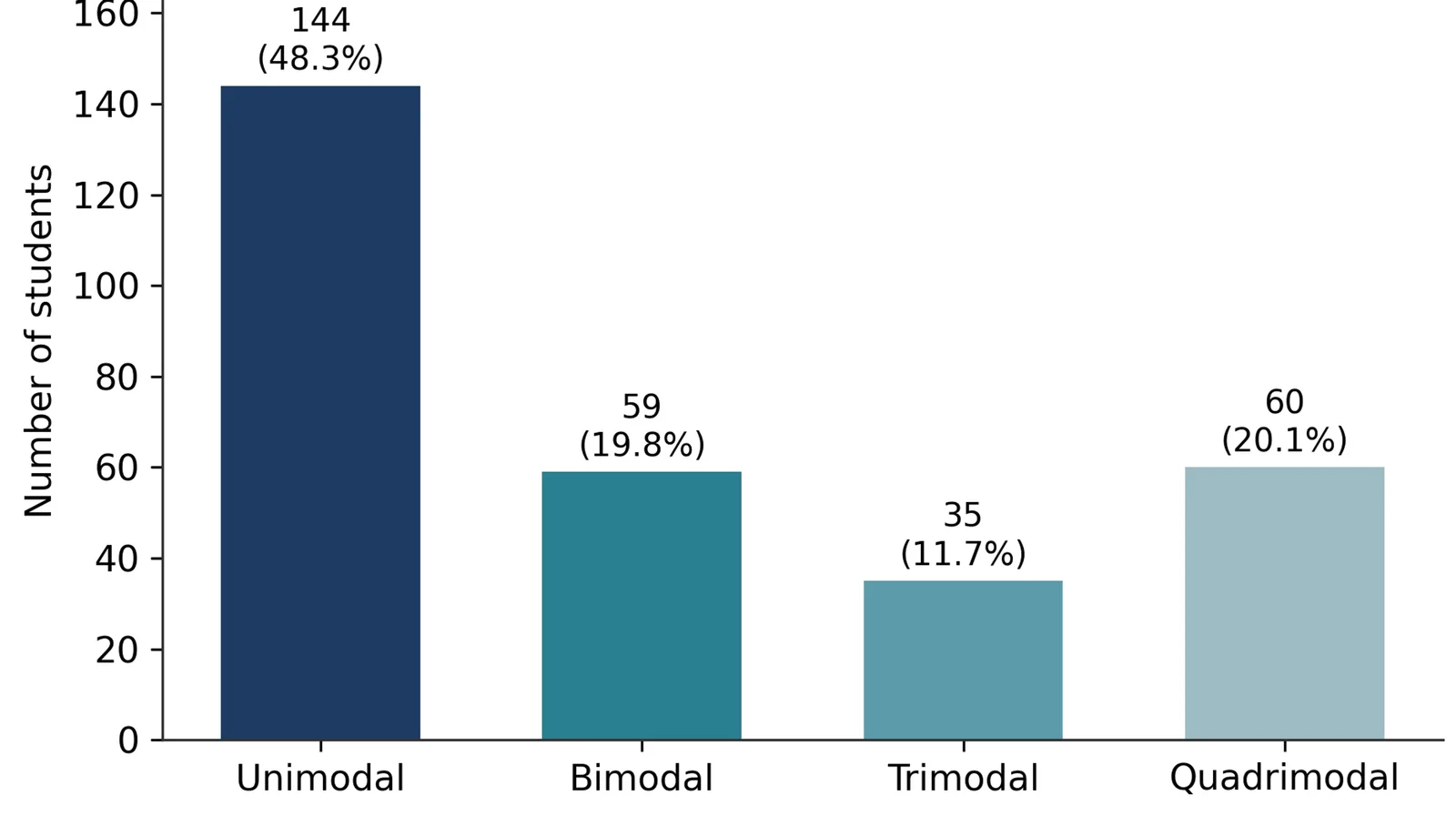
Distribution of VARK sensory-modality categories among undergraduate medical students (N=298) Values are numbers of students; percentages of the whole sample are shown above each bar. VARK: Visual, Aural, Read/write, Kinesthetic.

Among the 144 unimodal learners, kinesthetic preference clearly predominated, preferred by 112 students (77.8% of unimodal learners and 37.6% of the whole sample). Aural was the next most common single preference (19 students; 13.2% of unimodal learners), followed by visual (eight students; 5.6%) and read/write (five students; 3.5%) (Table 1).

**Table 1:** Sensory-modality preference among unimodal learners (n=144) Percentages were calculated using the total study sample (N=298) as the denominator.

Single modality (unimodal learners)	Students (n)	% of sample	% of unimodal
Kinesthetic (K)	112	37.6	77.8
Aural (A)	19	6.4	13.2
Visual (V)	8	2.7	5.6
Read/write (R)	5	1.7	3.5
Total unimodal	144	48.3	100

Within the multimodal group, the most common bimodal combination was aural-kinesthetic (AK; 42 students, 14.1% of the sample), followed by visual-kinesthetic (VK; eight), read/write-kinesthetic (RK; four), aural-read/write (AR; three), and visual-aural (VA; two). The most common trimodal pattern was visual-aural-kinesthetic (VAK; 23 students, 7.7%). Among the 60 quadrimodal (all-four) learners, 37 were classified as VARK Type Two, 16 as VARK Transition, and 7 as VARK Type One (Table 2).

**Table 2:** Multimodal preference combinations among the 154 multimodal learners V, visual; A, aural; R, read/write; K, kinesthetic. Percentages were calculated using the total study sample (N=298) as the denominator.

Multimodal combination	Students (n)	% of sample
Bimodal - AK	42	14.1
Bimodal - VK	8	2.7
Bimodal - RK	4	1.3
Bimodal - AR	3	1
Bimodal - VA	2	0.7
Trimodal - VAK	23	7.7
Trimodal - ARK	7	2.3
Trimodal - VRK	4	1.3
Trimodal - VAR	1	0.3
Quadrimodal - VARK Type Two	37	12.4
Quadrimodal - VARK Transition	16	5.4
Quadrimodal - VARK Type One	7	2.3

When raw scores were summed across all students, kinesthetic scored highest overall (total 2,690; mean 9.03) and read/write lowest (total 1,137; mean 3.82), with aural (total 1,964; mean 6.59) and visual (total 1,495; mean 5.02) intermediate (Table 3).

**Table 3:** Total and mean sensory-modality scores across all students (N=298) SD, Standard deviation.

Modality	Total score	Mean score	SD
Kinesthetic (K)	2,690	9.03	2.92
Aural (A)	1,964	6.59	3.12
Visual (V)	1,495	5.02	2.77
Read/write (R)	1,137	3.82	2.9

Gender-wise Comparison of Sensory-Modality Preferences

Statistical comparison of learning preferences and sensory-modality scores between men (n=139) and women (n=159) students. Value columns show counts (n) for categorical variables and means for continuous modality scores; SD columns apply only to the continuous scores. Categorical distributions were compared using the chi-square test (with Yates’ continuity correction) or Fisher’s exact test; modality scores were compared using the Mann-Whitney U test. A two-sided p-value <0.05 was considered statistically significant. Gender-wise comparisons of sensory-modality categories and individual modality scores are presented in Table 4.

**Table 4:** Comparison of sensory-modality preferences and scores by gender Values are presented as n (%) for categorical variables and mean±SD for continuous variables. A two-sided p<0.05 was considered statistically significant. SD, standard deviation; df, degrees of freedom; U, unimodal; B, bimodal; T, trimodal; Q, quadrimodal

Variable	Male (n=139)	Female (n=159)	Test	Statistic	df	p-value
							Unimodal vs. multimodal, n (%)			Chi-square (Yates’ correction)	3.75	1	0.053
Unimodal	76 (54.7%)	68 (42.8%)				
Multimodal	63 (45.3%)	91 (57.2%)				
Four-category distribution, n (%)			Chi-square	6.61	3	0.085
Unimodal	76 (54.7%)	68 (42.8%)				
Bimodal	24 (17.3%)	35 (22.0%)				
Trimodal	18 (12.9%)	17 (10.7%)				
Quadrimodal	21 (15.1%)	39 (24.5%)				
Visual score, mean±SD	4.88±2.51	5.14±2.99	Mann-Whitney U	10,726	-	0.66
Aural score, mean±SD	6.40±3.11	6.75±3.14	Mann-Whitney U	10,303	-	0.312
Read/write score, mean±SD	3.66±2.70	3.95±3.07	Mann-Whitney U	10,652	-	0.589
Kinesthetic score, mean±SD	8.92±3.06	9.12±2.76	Mann-Whitney U	10,722	-	0.657

A higher proportion of multimodal learners were female students (91 out of 159; 57.2%) compared to male students (63 out of 139; 45.3%). Fisher's exact test yielded p=0.048, whereas the chi-square test with Yates' continuity correction yielded p=0.053, indicating a borderline difference. The four-category distribution of unimodal, bimodal, trimodal, and quadrimodal preferences did not differ significantly by sex (χ²=6.61, df=3, p=0.085). Similarly, none of the four individual sensory-modality scores differed significantly between male and female students on Mann-Whitney U testing (all p>0.30) (Figure 2).

**Figure 2:**
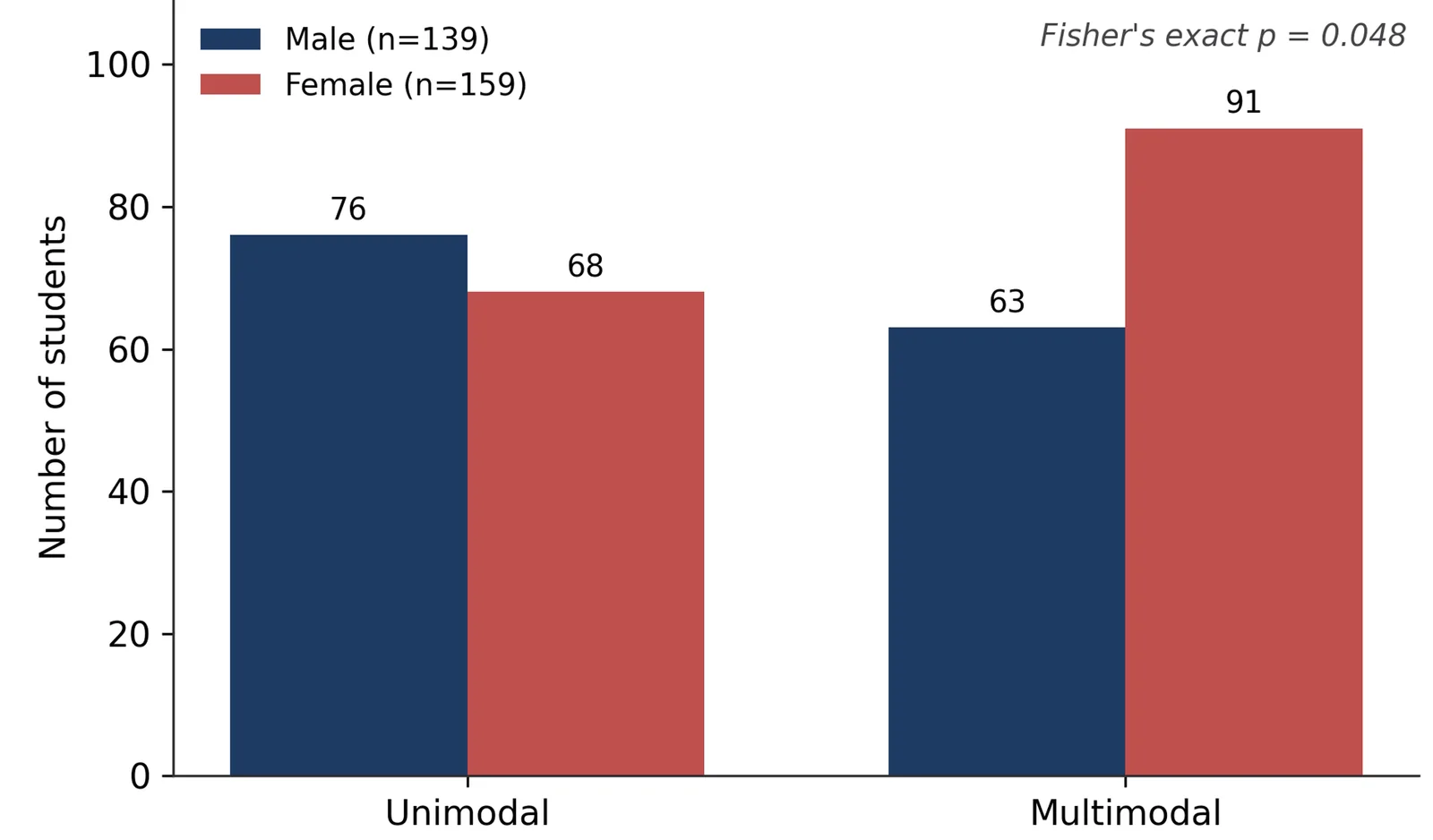
Unimodal versus multimodal preference by sex (N=298) Female students showed a higher proportion of multimodal learners than male students (57.2% vs. 45.3%; Fisher's exact p=0.048)

Comparison by MBBS Batch

Sensory-modality preferences were also examined across the four MBBS batches. The distribution of unimodal versus multimodal preferences did not differ significantly across batches (χ²=5.06, df=3, p=0.167). Similarly, the four-category distribution of unimodal, bimodal, trimodal, and quadrimodal preferences did not differ significantly across batches (χ²=12.68, df=9, p=0.177). Among the individual modality scores, visual, read/write, and kinesthetic scores did not differ significantly across batches. The aural score differed across batches (Kruskal-Wallis=10.09, df=3, p=0.018); Holm-adjusted pairwise analysis identified a significant difference between the 2019 and 2020 batches (adjusted p=0.017). The batch-wise results are presented in Table 5.

**Table 5:** Comparison of sensory-modality preferences and scores by MBBS batch Values are presented as n (%) for categorical variables and mean±SD for continuous variables. A two-sided p<0.05 was considered statistically significant. Categorical distributions were compared using the chi-square test. Continuous modality scores were compared using the Kruskal-Wallis test. Holm-adjusted pairwise Mann-Whitney U tests were performed following a significant Kruskal-Wallis result. SD, standard deviation; df, degrees of freedom.

Variable	2017 (n=75)	2018 (n=75)	2019 (n=75)	2020 (n=73)	Test	Statistic	df	p-value
Unimodal vs. multimodal, n (%)					Chi-square	5.06	3	0.167
Unimodal	42 (56.0%)	35 (46.7%)	29 (38.7%)	38 (52.1%)				
Multimodal	33 (44.0%)	40 (53.3%)	46 (61.3%)	35 (47.9%)				
Four-category distribution, n (%)					Chi-square	12.68	9	0.177
Unimodal	42 (56.0%)	35 (46.7%)	29 (38.7%)	38 (52.1%)				
Bimodal	13 (17.3%)	13 (17.3%)	14 (18.7%)	19 (26.0%)				
Trimodal	8 (10.7%)	11 (14.7%)	13 (17.3%)	3 (4.1%)				
Quadrimodal	12 (16.0%)	16 (21.3%)	19 (25.3%)	13 (17.8%)				
Visual score, mean±SD	5.23±2.81	5.04±2.88	5.19±2.87	4.60±2.53	Kruskal-Wallis	1.64	3	0.65
Aural score, mean±SD	6.44±3.01	6.44±3.15	7.52±3.13	5.95±3.06	Kruskal-Wallis	10.09	3	0.018
Read/write score, mean±SD	3.91±3.01	4.03±2.88	3.97±3.06	3.34±2.65	Kruskal-Wallis	2.5	3	0.476
Kinesthetic score, mean±SD	9.49±2.57	8.44±2.91	8.91±3.18	9.27±2.84	Kruskal-Wallis	4.6	3	0.204

## Discussion

Two findings stand out in this cohort of undergraduate medical students: a pronounced kinesthetic orientation and a near-even division between single-mode and multi-mode learners. The roughly equal split (48.3% unimodal, 51.7% multimodal) contrasts with several medical-student cohorts in which multimodal learners clearly predominate, for example, about 64% multimodal among first-year US medical students [9] and 86.8% at another Indian college [11], while resembling reports in which unimodal and multimodal learners occur in more comparable proportions.

The dominance of kinesthetic preference among single-mode learners is consistent with a recurring theme in the literature. Kinesthetic learning has emerged as the leading single modality in Turkish [20], Sri Lankan [21], and US [9] cohorts, and a kinesthetic-anchored multimodal profile has been described among students learning anatomy [22]. Although kinesthetic preference was the most common single-modality preference in our cohort, this finding does not establish that kinesthetic teaching is superior to other approaches. The VARK questionnaire describes students' reported preferences rather than the effectiveness of specific teaching modalities. Moreover, more than half of the students in our cohort had multimodal preferences. Therefore, the finding is better interpreted as supporting the inclusion of hands-on and experiential activities within a broader multimodal teaching approach rather than as evidence for adopting a predominantly kinesthetic teaching model. The present study was designed to describe sensory-modality preferences and did not evaluate whether these preferences were associated with academic performance or learning outcomes. Therefore, the findings cannot establish whether a particular sensory-modality preference is associated with better learning. Future prospective studies should examine VARK preferences alongside objective educational outcomes.

Read/write scored lowest on average in our sample, which is worth noting because online formats frequently rely heavily on text. It is important to be clear that this study did not assess learning outcomes or the effectiveness of any teaching format; the following considerations are therefore hypotheses generated by the preference data rather than conclusions about what works. With that caveat, a cohort oriented toward kinesthetic and aural input might engage less readily with delivery that is predominantly text-based, and pairing text with demonstrations, narrated presentations, virtual simulations, recorded procedural walk-throughs, and interactive tasks might align more closely with the group's expressed preferences [2,11]. Whether such alignment improves learning would need to be tested directly.

Female students preferred to be multimodal learners at a higher proportion than male students, although the statistical evidence was borderline (Fisher's exact p=0.048; Yates-corrected chi-square p=0.053) and was not reflected in the four-category distribution or mean modality scores. The wider literature is mixed: some studies report significant sex differences in learning-style preferences [16], others involving first-year medical cohorts find none [10,17], and still others describe sex-related differences in preferences and performance [18]. Given that the p-value was only marginally below the 0.05 threshold, that several comparisons were performed, and that neither the four-category distribution nor the mean modality scores differed by sex, this finding may reflect chance and should be regarded as exploratory only, to be confirmed or refuted in larger, multicenter samples. The exploratory comparison across MBBS batches did not demonstrate significant differences in the overall distribution of unimodal versus multimodal preferences or in the four-category distribution. Although the aural score differed across batches, the cross-sectional design prevents interpretation of this finding as a change in individual students' preferences over the course of medical education. Longitudinal studies following the same students across different stages would be needed to determine whether sensory-modality preferences change as students progress through the curriculum.

Importantly, describing a cohort's preferences does not imply that teaching to those preferences improves learning. Rigorous reviews have not supported the meshing hypothesis the idea that matching instruction to a learner's style enhances outcomes [12,14,15] and a study that examined whether students who studied in line with their VARK style performed better in anatomy found no such benefit [13]. We therefore interpret our results not as a mandate to sort students and match instruction to individual style labels, but as support for delivering content through multiple, active modalities that benefit the group as a whole.

This learner-centered approach is consistent with recent work in undergraduate medical education demonstrating the value of structured, interactive, and reflective learning approaches. The Joseph-RATA (rubrics ask-tell-ask) structured feedback model, for example, was associated with high learner acceptance, improved clarity of performance expectations, and promotion of self-directed learning, highlighting the potential value of active learner engagement within competency-based education [23]. On this basis, several practical strategies appear reasonable for a kinesthetically oriented cohort learning partly online: narrated slide decks and lecture recordings, early and frequent clinical exposure, the use of models, plastinates, and simulation, small-group discussion and seminars, and the deliberate pairing of text with visual and interactive material. Combining modalities in this way engages the range of preferences present in a cohort rather than privileging any single channel. Comparable recommendations to diversify teaching have followed VARK surveys of first-year medical students in other settings [24].

Limitations

This study has several limitations. It was conducted at a single center using a census of the accessible cohort; although all approached students responded, which minimizes non-response bias, the single-institution design limits generalizability to other settings. The data were based on self-report and may be subject to social-desirability and recall effects. The VARK questionnaire captures preference rather than ability, and its predictive validity for learning outcomes is debated [12,13]. Critically, we did not collect academic performance data or assess learning outcomes or the effectiveness of any teaching format; therefore, associations between sensory-modality preferences and academic performance or teaching effectiveness could not be examined. Finally, the cross-sectional design cannot establish causality or capture whether preferences change over time, and the borderline sex difference should be considered hypothesis-generating.

## Conclusions

Undergraduate medical students at this college showed a near-even distribution between unimodal and multimodal sensory-modality preferences, with kinesthetic preference being the most common single-modality preference. These findings describe the sensory-modality preferences reported by the cohort but do not demonstrate that any particular teaching modality is superior or more effective. Further studies linking sensory-modality preferences with educational outcomes are needed to determine their relevance to teaching and learning.
